# RNA-Targeted Therapeutics for Lipid Metabolic Disorders: From Bench to Bedside

**DOI:** 10.34133/research.1288

**Published:** 2026-05-19

**Authors:** Iqra Ali, Juhui Qiu, Wai San Cheang, Guixue Wang, Suowen Xu

**Affiliations:** ^1^ Key Laboratory for Biorheological Science and Technology of Ministry of Education, State and Local Joint Engineering Laboratory for Vascular Implants, College of Bioengineering, Chongqing University, Chongqing 400030, China.; ^2^ Institute of Panvascular Biology, JinFeng Laboratory, Chongqing 401329, China.; ^3^ State Key Laboratory of Mechanism and Quality of Chinese Medicine, Institute of Chinese Medical Sciences, University of Macau, Macau, SAR, China.; ^4^ Department of Endocrinology and Metabolism, Centre for Leading Medicine and Advanced Technologies of IHM, The First Affiliated Hospital of USTC, Division of Life Sciences and Medicine, University of Science and Technology of China, Hefei 230001, China.; ^5^ Anhui Provincial Key Laboratory of Metabolic Health and Panvascular Diseases, Hefei 230001, China.

## Abstract

Lipid accumulation drives the development of atherosclerotic cardiovascular disease, the leading cause of death worldwide. The liver is the primary target for lipid-lowering therapies due to its central role in lipid metabolism. Despite advances in small-molecule drugs, substantial residual cardiovascular risk remains. Recent clinical evidence in 2026 reaffirms the “lower is better” paradigm, showing that achieving very low low-density lipoprotein cholesterol (<55 mg/dl) improves outcomes versus standard goals (<70 mg/dl). RNA-targeted therapeutics, including antisense oligonucleotides (ASOs) and small interfering RNAs (siRNAs), offer a transformative approach by durably silencing genetically validated lipid targets. Conjugation to *N*-acetylgalactosamine (GalNAc) enables hepatocyte-specific delivery. Examples include inclisiran (targeting proprotein convertase subtilisin/kexin type 9 [PCSK9]) and agents against apolipoprotein C-III (APOC3), lipoprotein(a) [Lp(a)], and angiopoietin-like protein 3 (ANGPTL3). Beyond protein-coding messenger RNAs (mRNAs), RNA therapeutics can also target noncoding RNAs (microRNAs and long noncoding RNAs) that regulate cholesterol homeostasis. Additionally, mRNA lipid nanoparticle-based in vivo base editing (e.g., VERVE 101/102 targeting PCSK9) has entered clinical trials, offering potential for permanent genetic correction with a single infusion. In the future, RNA-targeted therapeutics may expand beyond hepatic lipid modulation to directly target vascular lipid metabolism and plaque biology. Advances in extrahepatic delivery and rational combination regimens could transform RNA therapy from transient lipid-lowering to long-term, disease-modifying interventions. The recent 2026 American College of Cardiology/American Heart Association dyslipidemia guideline emphasizes the clinical importance of targeting PCSK9, APOC3, Lp(a), and ANGPTL3. This review provides a state-of-the-art overview of RNA-targeted therapeutics in hyperlipidemia and discusses future research directions in this emerging field.

## Introduction

**Fig. 1.**
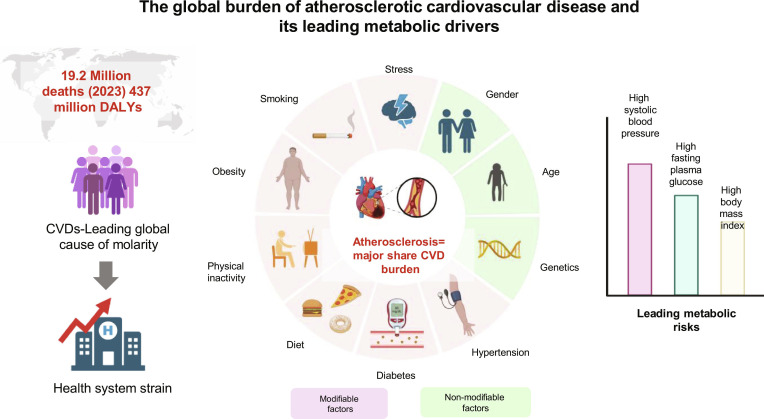
The global burden of atherosclerotic cardiovascular disease (ASCVD) and its leading metabolic drivers. This schematic summarizes the epidemiology and key modifiable risk factors for CVD. In 2023, CVDs caused an estimated 19.2 million deaths and 437 million disability-adjusted life years (DALYs) globally, with atherosclerotic conditions representing the predominant share. In addition to nonmodifiable factors such as age, genetics, and sex, the burden is largely driven by modifiable metabolic risks, primarily high systolic blood pressure, high fasting plasma glucose, and high body mass index, which collectively underscore the shift toward comprehensive metabolic regulation in ASCVD prevention.

Lipid metabolic disorders are major global health challenges and represent the key drivers of atherosclerotic cardiovascular disease (ASCVD), which continues to be the primary cause of death globally [1,2]. Cardiovascular diseases (CVDs) were responsible for 437 million disability-adjusted life years (DALYs) and an expected 19.2 million deaths in 2023, with the clinical manifestations of atherosclerosis constituting the largest share of this burden [3–5]. Critically, approximately 80% of the attributable CVD burden is linked to modifiable risk factors [5]. The principle that “lower is better” for low-density lipoprotein cholesterol (LDL-C) has recently been reinforced by a randomized trial showing that achieving LDL-C levels below 55 mg/dl substantially reduces major cardiovascular events compared with the standard target of below 70 mg/dl (hazard ratio, 0.67; 95% CI, 0.52 to 0.86; *P* = 0.002) (NCT04626973) [6]. A focused analysis of the metabolic burden revealed that while high systolic blood pressure remains a major contributor, the increasing impact of metabolic risk, like elevated plasma glucose during fasting and high body mass index (BMI), highlights the need for strategies targeting lipid and metabolic dysregulation (Fig. 1) [7].

ASCVD arises as a downstream consequence of chronic disturbances in lipid metabolism [8,9]. Nowadays, it is increasingly recognized as a lipid-inflammatory disorder, where substantial residual inflammatory risk persists even in optimally lipid-controlled patients, underscoring the need for therapeutic strategies that address both drivers of atherosclerosis [10]. Its lipid-driven inflammatory [11] process is initiated by endothelial dysfunction [12], which allows apolipoprotein B (ApoB)-containing lipoproteins [13], primarily LDL [14,15] and very-low-density lipoprotein (VLDL) remnants, to infiltrate the arterial intima. Within the subendothelial space, these particles undergo oxidative modification [16], triggering a pathogenic cascade: endothelial cells express adhesion molecules that recruit monocytes, which develop into macrophages and become foam cells by consuming modified lipids, contributing to early lesion formation and eventually to ASCVD [17]. This process escalates as activated immune cells and vascular smooth muscle cells promote and facilitate plaque development [18], which is distinguished by a lipid-rich necrotic core and a fibrous cap [19].

Despite decades of therapeutic advances targeting this pathway, such as the optimum reduction of LDL-C using statins, ezetimibe, bempedoic acid, or proprotein convertase subtilisin/kexin type 9 (PCSK9) monoclonal antibodies, a considerable residual cardiovascular risk remains [20,21]. Genetically determined lipoproteins and regulatory proteins, notably PCSK9, lipoprotein(a) [Lp(a)], apolipoprotein C-III (ApoC3), and angiopoietin-like protein 3 (ANGPTL3), are now recognized as major, independent causal drivers of ASCVD [22–26]. Lp(a), an LDL-like particle linked to the unique apolipoprotein(a), promotes plaque inflammation [27,28] and calcification [29]. ApoC3 delays the elimination of triglyceride (TG)-rich lipoproteins, resulting in the buildup of atherogenic remains [30–33]. ANGPTL3 regulates lipid metabolism through the inhibition of lipoprotein lipase and endothelial lipase [34,35], and its production is dictated at the transcriptional level in hepatocytes [36]. Familial hypercholesterolemia (FH) is a prevalent inherited lipid condition caused mostly by LDL receptor (LDLR) mutations and, less commonly, by PCSK9 or ApoB variants, and exemplifies the clinical consequences of dysregulated lipoprotein metabolism. Individuals with FH experience lifelong elevations of LDL-C and substantially higher chance of premature ASCVD, highlighting the translational importance of targeting these genetic pathways [37].

The treatment of CVDs, including ASCVD, has shifted toward drugs that target specific pathways, such as receptors and enzymes [38]. We are now entering a new era with novel advanced therapies (NATs), particularly RNA-targeted therapeutics [39], a term we use here to refer specifically to antisense oligonucleotides (ASOs) and small interfering RNAs (siRNAs) that achieve gene silencing through sequence-specific binding to target RNA transcripts (including both protein-coding messenger RNAs [mRNAs] and noncoding RNAs [ncRNAs]). These treatments work by silencing the expression of single genes that cause disease, exhibiting a more precise and genetically informed strategy for managing ASCVD. Despite these advances, residual cardiovascular risk persists, motivating developing innovative medicines that directly target the genetic underpinnings of lipid dysregulation. Among the most modern therapies in the cardiovascular area are ASOs and siRNAs [38], which achieve highly specific gene silencing via attaching to and causing the degradation of target mRNAs [40,41]. This approach requires only sequence complementarity, enabling the precise inhibition of previously inaccessible pathogenic transcripts [42]. The clinical viability of this platform has been revolutionized by *N*-acetylgalactosamine (GalNAc) conjugation, which allows for targeted distribution to hepatocytes, the primary site of synthesis for these atherogenic proteins via the asialoglycoprotein receptor (ASGPR) [43,44]. The clinical validation of this paradigm was achieved with inclisiran, a GalNAc-conjugated siRNA targeting PCSK9, demonstrating a consistent LDL-C decrease with 2-year dosage [45].

This review highlights how RNA-targeted therapeutics are transforming the management of lipid-driven diseases and their downstream cardiovascular consequences, including ASCVD, by targeting their genetic causes. We describe key targets, PCSK9, Lp(a), ApoC3, and ANGPTL3, and why RNA platforms are well suited to modulate them. Finally, we summarize the progression from preclinical studies to late-stage trials, illustrating the shift toward gene-level interventions. While previous reviews have provided excellent summaries of RNA-targeted therapies for dyslipidemia, including the foundational work by Tsimikas [46] and the recent comprehensive analysis by Kosmas et al. [47], the present manuscript offers an updated perspective. Unlike these prior reviews, which focused exclusively on protein-coding mRNAs and ASO/siRNA silencing, we expand the scope to include (a) ncRNAs (miRNAs and lncRNAs) as targets of RNA-targeted therapeutics, with detailed preclinical evidence for anti-miR ASOs, and (b) mRNA-LNP-based in vivo base editing (e.g., VERVE-101 and VERVE-102 targeting PCSK9), a rapidly emerging modality that has reached clinical trials. In addition, we incorporate the most recent clinical developments and guidelines (e.g., ORION-16, GATEWAY, the 2026 American College of Cardiology [ACC]/American Heart Association [AHA] guideline, and the 2026 ACC Scientific Statement on Gene Editing Therapy in Cardiovascular Disease) and briefly discuss emerging modalities that extend beyond traditional ASOs and siRNAs, such as CRISPR-mediated genome editing, which is RNA-based but not RNA-targeted (as it targets DNA rather than binding directly to a disease-causing mRNA). Together, these elements provide a timely and distinct contribution to the field.

## Established Therapies for the Management of Lipid-Metabolic Disorders

Statins, which inhibit 3-hydroxy-3-methylglutaryl-coenzyme A (HMG-CoA) reductase, remain the first-line therapy for lowering LDL-C by 30% to 50% and reducing cardiovascular events across diverse populations [48,49]. Their mechanism of action involves blocking the speed-limiting stage in hepatic cholesterol production, which up-regulates LDLRs and enhances LDL clearance [50]. Despite proven efficacy, statin monotherapy frequently fails to achieve contemporary lipid targets in real-world practice. The European DA VINCI registry reported that while 84% of patients on lipid-lowering therapy received statin monotherapy, only 33% attained the 2019 European Society of Cardiology/European Atherosclerosis Society (ESC/EAS) LDL-C goals [51,52]. The benefits of statins extend across age groups, with a large target trial emulation confirming that initiating statins for primary prevention considerably reduces cardiovascular risk in adults aged 75 to 84 years and even in those aged 85 years and older [53]. Additionally, statin use has been associated with reduced risk of chronic liver disease and hepatocellular carcinoma, providing another rationale for its use in at-risk populations [54].

Ezetimibe is a second-line pharmacological treatment for patients at risk of ASCVD, either as add-on to statin therapy or as monotherapy (NCT00202878) [55–57]. It lowers cholesterol by inhibiting intestinal and biliary absorption, reducing both LDL-C and non-HDL-C [58]. Oral ezetimibe (10 mg daily) reduces Lp(a) by approximately 7%, though this is of limited clinical relevance [59,60]. The RACING trial demonstrated that combining moderate-intensity rosuvastatin with ezetimibe was noninferior to high-intensity rosuvastatin monotherapy for 3-year cardiovascular outcomes, with superior LDL-C control and better tolerability (NCT03044665) [61]. The Improved Reduction of Outcomes: Vytorin Efficacy International trial demonstrated that for patients experiencing acute coronary syndrome, the incorporation of ezetimibe to intense statin therapy resulted in a substantial decrease in major cardiovascular events. This benefit was particularly pronounced in the subgroup of individuals who also had diabetes mellitus [55,62].

Bempedoic acid is a new cholesterol-lowering drug that inhibits ATP-citrate lyase, an enzyme that operates faster in the cholesterol production pathway than HMG-CoA reductase, the target of statins [63]. In the CLEAR Outcomes trial, treatment in statin-intolerant patients resulted in a 21.1% greater LDL-C reduction and a 13% lower risk of major adverse cardiovascular events (MACE) versus placebo, with no excess muscle-related adverse events but higher incidences of gout and cholelithiasis [64]. A prespecified analysis of 4,206 primary prevention patients demonstrated even greater relative risk reduction for the primary cardiovascular endpoint (hazard ratio [HR] 0.70) and significant reductions in cardiovascular and all-cause mortality (NCT02993406) [65]. Furthermore, supported by phase 3 evidence, bempedoic acid is approved for use either alone in statin-intolerant individuals or as supplementary therapy in combination with statins and ezetimibe in patients with elevated risk of FH or ASCVD who have not reached their desired LDL-C targets (NCT05103254) [66].

Evolocumab and alirocumab (PCSK9 monoclonal antibodies) bind circulating PCSK9, preventing it from degrading LDLRs on hepatocytes, thereby increasing LDL-C clearance [67–69]. In the FOURIER trial, evolocumab reduced LDL-C by 59% and decreased MACE in ASCVD patients (HR 0.85) (NCT01764633) [70]. In the ODYSSEY OUTCOMES trial, alirocumab added to statin therapy reduced LDL-C by 54.7% compared with placebo (from 101.4 to 53.3 mg/dl) and significantly decreased MACE (HR 0.85; 95% CI 0.78 to 0.93) (NCT01663402) [71]. Recent meta-analyses confirm that both agents significantly reduce myocardial infarction, stroke, and coronary revascularization, with some outcome-specific differences between the 2 drugs [72,73]. Beyond LDL-C lowering, PCSK9 inhibitors also reduce Lp(a) by 20% to 30%, supporting their use in patients with elevated Lp(a) as reflected in the 2023 ESC guidelines (Class IIa recommendation) [74]. These agents are generally reserved for high-risk patients not achieving LDL-C goals on maximally tolerated statin and ezetimibe therapy, or those with statin intolerance [75]. More recently, tafolecimab has been approved in China as a third PCSK9 monoclonal antibody, demonstrating robust LDL-C reductions of over 60% in clinical trials [76]. Furthermore, the oral small-molecule PCSK9 inhibitor enlicitide decanoate was evaluated in the phase 3 CORALreef Lipids trial (NCT05952856). In 2,909 patients with or at risk for ASCVD, enlicitide 20 mg daily reduced LDL-C by 57.1% at 24 weeks compared with placebo (*P* < 0.001), with significant improvements in non-HDL-C, ApoB, and Lp(a) and a favorable safety profile [77].

Fibrate primarily modulates the lipid profile by decreasing TGs and increasing high-density lipoprotein cholesterol [78]. Observational data have suggested a potential cardiovascular benefit of fibrates in patients with chronic kidney disease (CKD), with a nested case–control study reporting an association between fibrate use, particularly pemafibrate, and a lower risk of MACE [79]. A prespecified subgroup analysis of the ACCORD-LIPID trial indicated that fenofibrate improved cardiovascular outcomes, specifically in individuals with atherogenic dyslipidemia, as characterized by high TGs (≥204 mg/dl) and low HDL-C (<34 mg/dl). This benefit was not observed in the broader group of type 2 diabetes patients treated with simvastatin (NCT00000620) [80]. The PROMINENT outcomes trial demonstrated that although pemafibrate improved atherogenic lipid parameters, it did not translate into a reduction in CVDs for high-risk patients with type 2 diabetes and mixed dyslipidemia (NCT03071692) [81].

Icosapent ethyl (IPE), a highly purified ethyl ester of eicosapentaenoic acid (EPA), was first investigated for its efficacy and safety in clinical trials, such as the MARINE (NCT01047683) [82] and ANCHOR studies (NCT01047501) [83]. IPE (4 g/day) significantly reduced MACE by 25% in the REDUCE-IT trial, with consistent benefit even in patients with baseline LDL-C <55 mg/dl. Cardiovascular death was reduced by 20%, though therapy was associated with increased atrial fibrillation risk [84,85]. However, the use of mineral oil as the placebo comparator has generated discussion regarding potential effects on lipid and inflammatory biomarkers in the control group [86]. Despite this debate, IPE has been incorporated into contemporary lipid management strategies such as AHA and ACC guidance generally recommends it as Class IIb [87], while lipid-focused societies such as the National Lipid Association (NLA) makes a Class I recommendation for use of IPE (4 g/d) for selected high-risk populations [88]. However, its moderate absolute benefit, requirement for high daily dosing, and atrial fibrillation risk highlight limitations of small-molecule approaches.

While the small-molecule drugs discussed above are effective at lowering lipids and mitigating cardiovascular risk, they have inherent limitations that necessitate the development of novel therapies, such as NATs. Drugs such as statins, ezetimibe, and bempedoic acid have a brief duration of action and must be taken every day. While consistent use is essential for reducing LDL cholesterol effectively, real-world adherence to statin therapy is frequently poor, and the duration of treatment decreases [63,89]. Nucleic acid-based therapies may provide greater therapeutic alternatives that try to fix the genetic problem at its root by changing and correcting the disease-causing gene [90]. However, this novel modality is not without its own set of challenges. The widespread adoption of NATs will depend on overcoming substantial hurdles, including high manufacturing costs that may limit patient access (with some genetic therapies exceeding $4 million per patient) [91], lingering questions regarding long-term safety that require ongoing investigation [92], and the risks of immunogenicity and off-target effects that have caused clinical trial failures [93]. Furthermore, real-world implementation demands new healthcare infrastructure, coordination across specialties, and navigation of insurance coverage barriers [94,95]. These pharmacological and practical considerations underscore that NATs are poised to complement, rather than entirely replace, small-molecule therapies.

## Emerging RNA-Targeted Therapeutics for ncRNAs

In addition to targeting protein-coding mRNAs, ASO and siRNA platforms can be directed against ncRNAs, including microRNAs (miRNAs) and long noncoding RNAs (lncRNAs). This remains an RNA-targeted strategy, as the therapeutic agent binds directly to the ncRNA transcript. Although most of these approaches are still in preclinical or early clinical stages, they represent important future directions for RNA-targeted therapeutics in dyslipidemia and atherosclerosis.

### ncRNAs as therapeutic targets

ncRNAs, including miRNAs and lncRNAs, regulate gene expression without encoding proteins and have been implicated in the pathogenesis of dyslipidemia and atherosclerosis [96–98]. Among the most studied miRNAs are miR-33a/b**,** which are intronic miRNAs embedded within the sterol regulatory element-binding protein genes *SREBF2* and *SREBF1*. These miRNAs act as key posttranscriptional repressors of the cholesterol transporter ABCA1, thereby limiting HDL biogenesis and reverse cholesterol transport [99,100]. Antisense inhibition of miR-33a/b in nonhuman primates increases hepatic ABCA1 expression, raises plasma HDL cholesterol, and lowers VLDL-associated TGs [101], while genetic deletion of miR-33 in mice elevates HDL levels [100]. Beyond lipid metabolism, miR-33a/b also regulates fatty acid oxidation and insulin signaling [102,103]. Despite the therapeutic promise of miR-33 antagonism for dyslipidemia and atherosclerosis, systemic inhibition requires caution due to potential effects on hunger signaling and obesity [103].

miR-122 is a liver-specific miRNA that critically regulates cholesterol and lipid homeostasis. Inhibition of miR-122 reduces plasma cholesterol and hepatic cholesterol synthesis rates [104], whereas genetic deletion leads to hepatosteatosis and dyslipidemia [105]. miR-122 also regulates cholesterol catabolism by suppressing *Cyp7a1* [106] and modulates hepatic lipid metabolism through pathways involving fatty acid synthesis and peroxidation [107]. miR-148a is a key posttranscriptional regulator of LDLR expression, directly targeting the LDLR 3′UTR to reduce hepatic LDL-C clearance [108]. It also indirectly suppresses LDLR by repressing the (pro)renin receptor [(P)RR], which stabilizes LDLR protein [109]. Beyond LDL metabolism, miR-148a inhibits ABCA1, thereby limiting HDL biogenesis and reverse cholesterol transport. Therapeutic silencing of miR-148a in mice increases hepatic LDLR and ABCA1 expression, lowering LDL-C while raising HDL-C [108,110], and reduces atherosclerotic plaque burden and promotes plaque stability [111]. Recent nanoparticle-based strategies have been developed to deliver miR-148a inhibitors for hyperlipidemia therapy [112]. Thus, miR-33a/b, miR-122, and miR-148a each play distinct but complementary roles in controlling cholesterol homeostasis, highlighting the therapeutic potential of targeting these miRNAs for the management of dyslipidemia and atherosclerosis.

The therapeutic targeting of miRNAs is achieved using anti-miRNA oligonucleotides (anti-miRs or antagomirs), which are chemically modified ASOs that bind directly to the mature miRNA and block its function [113]. Preclinical “bench: evidence for this approach is robust. Anti-miR-33 treatment in Ldlr^−/−^ mice with established atherosclerotic plaques increased HDL-C, enhanced reverse cholesterol transport, reduced plaque size and lipid content, and increased markers of plaque stability [114]. Anti-miR-122 silencing in mice lowered plasma cholesterol levels and improved hepatic steatosis [104], and the landmark antagomir study demonstrated that miR-122 inhibition reduces cholesterol biosynthesis gene expression and plasma cholesterol [113]. Furthermore, GalNAc-conjugated anti-miR-122 (tiny locked nucleic acid) achieved ~300- to 500-fold greater potency than unconjugated counterparts, illustrating the translational potential of liver-targeted anti-miR delivery [115]. For miR-148a, anti-miR-148a silencing in mice increases hepatic LDLR and ABCA1 expression, lowers LDL-C while raising HDL-C, and reduces atherosclerotic plaque burden [108,111]. Collectively, these studies establish anti-miR therapeutics as a major RNA-targeted strategy for lipid disorders, with strong preclinical evidence supporting their continued development.

In addition to miRNAs, lncRNAs have emerged as important regulators of lipid metabolism and cholesterol homeostasis. LncRNA LeXis is induced by liver X receptor (LXR) activation or a Western diet and mediates the inhibition of cholesterol biosynthesis by interacting with the RNA-binding protein RALY (heterogeneous nuclear ribonucleoprotein) [116]. Its human orthologue (TCONS_00016452) has been proposed as a noninvasive diagnostic biomarker for nonalcoholic steatohepatitis [117], and LeXis also contributes to cholesterol efflux by modulating ABCA1 expression [118,119]. Although research is still evolving, LeXis represents a promising lncRNA target for future RNA-targeted therapies against dyslipidemia and atherosclerosis [120,121].

MeXis is an lncRNA that amplifies LXR-dependent transcription of the cholesterol efflux transporter *Abca1* by interacting with the transcriptional coactivator DDX17. Loss of MeXis alters chromatin architecture at the *Abca1* locus, impairs cholesterol handling, and accelerates atherosclerosis in mice [122]. Accordingly, MeXis is recognized as one of several lncRNAs that regulate ABCA1-mediated cholesterol efflux and atherosclerosis development [118]. CHROME is a primate-specific lncRNA whose expression is induced by dietary cholesterol via LXR activation and is elevated in the plasma and atherosclerotic plaques of patients with coronary artery disease. It promotes cholesterol efflux and HDL biogenesis by sponging miR-27b, miR-33a/b, and miR-128, thereby derepressing their target gene *ABCA1* [123]. Consistent with a role in lipid regulation, CHROME expression is up-regulated by atorvastatin treatment in hypercholesterolemic patients, suggesting its involvement in the response to statin therapy [124]. Together, these findings position CHROME as a key regulator of cholesterol homeostasis and a potential therapeutic target for dyslipidemia and atherosclerosis [119]. Collectively, these miRNAs and lncRNAs constitute a complex regulatory network that controls cholesterol homeostasis, and their therapeutic modulation holds promise for the management of dyslipidemia and atherosclerosis.

### RNA-based in vivo base editing: mRNA-LNP delivery for PCSK9 inactivation

Beyond RNA-targeted gene silencing, the therapeutic landscape has expanded to include mRNA-based in vivo base editing, which represents a paradigm shift from transient protein modulation to permanent genetic correction [125]. This approach uses lipid nanoparticles (LNPs) to deliver 2 RNA components: an mRNA encoding an adenine base editor and a guide RNA (gRNA) targeting a specific genomic locus. Upon hepatocyte uptake, the base editor introduces a precise single-base change in the DNA, permanently inactivating the target gene without double-strand breaks [125,126].

This strategy has been most clinically advanced for PCSK9 inactivation. VERVE-101, the first in vivo base editing therapy to enter clinical trials (NCT05398029), consists of an adenine base editor mRNA and an optimized gRNA targeting *PCSK9*, packaged in an LNP. In a nonhuman primate study, a single intravenous infusion of VERVE-101 (1.5 mg/kg) achieved 70% PCSK9 editing in the liver, resulting in 83% reduction in circulating PCSK9 protein and 69% reduction in LDL-C, with durable effects lasting up to 476 d. Transient liver enzyme elevations resolved without sequelae, and no germline editing was detected [127]. Based on these data, VERVE-101 advanced to the phase 1b Heart-1 trial in patients with heterozygous FH and established ASCVD [128].

VERVE-102 (NCT06164730) is a next-generation base editing therapy that uses a GalNAc-conjugated LNP (GalNAc-LNP) to achieve more hepatocyte-specific delivery. This design enables hepatocyte uptake via either the LDLR or the ASGPR, potentially overcoming reduced LDLR function in FH [129]. In nonhuman primates, a single infusion of VERVE-102 (3 mg/kg) led to durable mean reductions of 80% in blood PCSK9 and 62% in LDL-C. Off-target editing was not detected across a panel of ~6,000 candidate sites, and no germline transmission was observed [130]. The ongoing phase 1b Heart-2 trial is evaluating safety, tolerability, and pharmacodynamics in patients with heterozygous familial hypercholesterolemia or premature coronary artery disease [129,130]. These clinical milestones demonstrate that mRNA-LNP delivery for in vivo base editing has moved from bench to bedside for lipid disorders, complementing RNA-targeted silencing approaches. However, long-term safety, off-target editing risks, and durability beyond the lifespan of edited hepatocytes remain important considerations [125,126,128]. The 2026 ACC Scientific Statement on Gene Editing Therapy in Cardiovascular Disease highlights VERVE-102 as a leading example of in vivo base editing for lipid disorders, noting interim LDL-C reductions of 53% to 69% and emphasizing the potential for durable, single-infusion treatment [131].

## Potential Advantages of RNA-Targeted Therapeutics

Managing cholesterol to prevent ASCVD is a long-term commitment, so any successful treatment must be both safe and easy to tolerate over many years. While standard daily medications such as statins are effective in principle, they depend on consistent daily use to maintain their benefit. In practice, many patients find it hard to adhere to a daily routine, and adherence often decreases over time, undercutting the treatment’s real-world impact. This reliance on perfect adherence further underscores the importance of safety and tolerability [63,89]. For these reasons, research is now shifting toward innovative treatments such as RNA-targeted therapeutics (siRNA and ASO therapies). This strategy marks a breakthrough advance in medicine, enabling the precise targeting of molecules and pathways that traditional drugs and antibody treatments could not previously address. In their development and study, computational methods, especially molecular docking [132] and molecular dynamics simulations [133], are essential tools.

The 2 primary categories of these RNA-targeting drugs are ASOs and siRNAs. Clinically, the key distinction lies in durability: siRNAs achieve prolonged effects through catalytic recycling within RNA-induced silencing complex, whereas ASOs are not recycled and typically require more frequent administration [134–136]. This difference in pharmacological durability was illustrated in the race to develop PCSK9-targeting RNA therapies. The GalNAc-conjugated ASO AZD8233, while effective, required monthly dosing to maintain LDL-C reduction. Its development was discontinued in 2022 [137], whereas the siRNA (inclisiran) achieved approval with a 2-year regimen, underscoring how dosing convenience and sustained efficacy have become decisive factors for chronic ASCVD management. siRNAs can be created artificially and treated, or they can form naturally inside cells from larger RNA molecules, such as those produced during a viral infection [138].

**Fig. 2.**
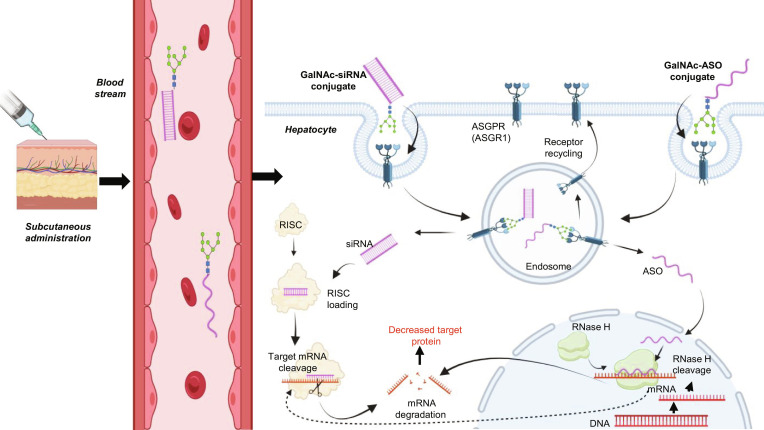
This figure illustrates the mechanism of liver-targeted gene silencing by subcutaneously administered *N*-acetylgalactosamine (GalNAc)-conjugated oligonucleotides. The GalNAc ligand directs both the small interfering RNA (siRNA) and antisense oligonucleotide (ASO) conjugates to hepatocytes by binding to the ASGPR (also known as ASGR1) and subsequent endocytosis. For siRNA (left), the released RNA strand loads into the RISC complex to guide sequence-specific cleavage and degradation of the target mRNA, reducing protein expression. For the ASO (right), the DNA-based oligo hybridizes with target mRNA, recruiting RNase H to cleave the mRNA, enabling catalytic reuse of the ASO. Both pathways result in potent gene silencing, with ASGPR recycled to the cell surface.

RNA-targeted approaches are inherently modular [139,140]. The therapeutic potential of siRNA-targeted therapeutics has been established across a wide range of disorders, with numerous siRNA-based pharmaceuticals already available on the market, such as inclisiran to reduce LDL-C levels in patients with ASCVD [141], and patisiran to treat hereditary transthyretin-mediated amyloidosis [142]. The development of GalNAc-siRNA technology is pivotal for RNA interference (RNAi) treatment [143]. GalNAc ligands bind exceptionally high affinity to the ASGPR, a cell-surface receptor expressed almost exclusively on hepatocytes. This selective interaction facilitates efficient, receptor-mediated endocytosis of GalNAc-conjugated RNA molecules following simple subcutaneous injection (Fig. 2) [144–148]. The liver is the central organ for the synthesis of ApoB-containing lipoproteins and Lp(a), as well as hepatocyte-derived regulators such as ApoC3, ANGPTL3, and PCSK9; therefore, hepatocyte-specific delivery aligns perfectly with the molecular origins of atherosclerotic risk [33,149–153].

**Fig. 3.**
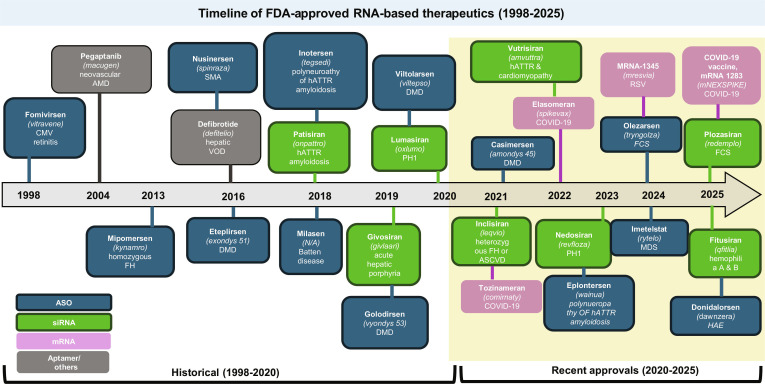
Timeline of U.S. Food and Drug Administration (FDA)-approved RNA-based therapeutics (1998 to 2025). Chronological overview of selected RNA-based therapies granted approval by the FDA from 1998 to 2025. Entries are organized by approval year and list the therapeutic agent alongside its primary indication.

FDA-approved RNA-targeted therapies span a variety of indications (Fig. 3), demonstrating the versatility and clinical maturity of this platform. The first approved RNA drug, fomivirsen (ASO, 1998), targeted cytomegalovirus retinitis, establishing early proof of concept for oligonucleotide therapeutics [154]. Since then, RNA-based drugs have been approved for diverse conditions including macular degeneration (pegaptanib and aptamer, 2004) [155]. Eteplirsen (ASO), which was also approved in 2016, is used for Duchenne muscular dystrophy [156]. Givosiran (2019) was indicated for acute hepatic porphyria [157]. Lumasiran (2020) is FDA approved for primary hyperoxaluria type 1 (PH1) [158]. Nedosiran (2023) is an FDA-approved method for lowering urinary oxalate levels in specific patients with PH1, a rare genetic disorder [159]. Donidalorsen (2025) is FDA-approved for the prevention of hereditary angioedema (HAE) attacks [160].

For lipid disorders specifically, several RNA-targeted therapies have now been approved. Mipomersen (ASO), approved in 2013, targets FH [161], whereas defibrotide (a complex mixture of oligonucleotides, 2016) is indicated for hepatic veno-occlusive disease [162]. Inclisiran (2021) became the first small-interfering RNA therapy for heterozygous FH or clinical ASCVD [163]. More recently, plozasiran (2025) received FDA approval for treating familial chylomicronemia syndrome (FCS) [164]. The successful development of these lipid-targeting RNA therapies has paved the way for investigational candidates such as pelacarsen (ASO) and olpasiran (siRNA), which are currently in clinical trials for lowering Lp(a) and addressing residual cardiovascular risk. The potential impact of these therapies extends beyond lowering lipid levels [165]. RNA platforms offer the ability to modulate inflammatory mediators [166,167], vascular remodeling pathways, and perhaps plaque instability itself [168]. RNA-targeted therapeutics not only represent incremental additions to existing therapies but also constitute a new class of gene-directed medicines capable of addressing the root causes of atherosclerosis. By expanding the range of druggable targets and providing durable, hepatocyte-specific modulation of pathogenic pathways, RNA-targeted therapeutics hold the potential to redefine lipid-driven ASCVD management.

## RNA-Targeted Therapeutics for Lipid-Metabolic Disorders: Bench Evidence

The development of RNA-targeted therapeutics is built on strong preclinical evidence of their unique mechanism. By silencing genes before protein translation, ASOs and siRNAs overcome the transient nature and protein-targeting limitations of small molecules. This is exemplified by the targeting of key hepatic genes [*PCSK9*, *APOC3*, *Lp(a)*, and *ANGPTL3*], whose loss of function in models confers substantial atheroprotection without harming cells, confirming their suitability for RNA-targeted therapy. This therapeutic promise was realized through parallel breakthroughs in delivery technology.

Advances in the design and delivery, most notably GalNAc conjugation for hepatocyte targeting and the development of LNPs, have enabled the efficient hepatic delivery of these therapeutics, leading to the rapid development of NATs [38,169]. Critical to this progress has been the ongoing optimization of the GalNAc platform itself. Compared with the traditional triantennary structure, a novel serial monovalent GalNAc conjugate design featuring strategically spaced units provided superior in vivo potency and a longer duration of action in mice. This optimization, which is linked to improved intracellular stability, highlights the active engineering of the platform itself to enhance pharmacokinetics and therapeutic efficacy [170]. The success of this liver-targeted paradigm, pioneered in cardiovascular disease by the GalNAc-conjugated siRNA inclisiran, has established it as the foundational and most clinically advanced strategy, even as efforts continue to achieve efficient delivery to other cardiovascular tissues [171,172].

The translational potential of RNAi for cholesterol management was decisively demonstrated by seminal preclinical research. A cross-species, LNP-formulated siRNA targeting *PCSK9* induced potent (50% to 70%) and durable silencing of hepatic mRNA in rodents and nonhuman primates. This resulted in a sustained reduction in plasma PCSK9 protein and a corresponding >60% decrease in LDL cholesterol that lasted several weeks after a single dosage, without affecting HDL-C or TGs. Furthermore, preclinical models have demonstrated that PCSK9 silencing concurrently reduces inflammatory pathways, including the down-regulation of key hepatic proinflammatory signaling cascades such as Toll-like receptors, mitogen-activated protein kinase (MAPK), and phosphoinositide 3-kinase/protein kinase B (PI3K/Akt), thereby attenuating downstream nuclear factor kappa-light-chain-enhancer of activated B cells (NF-κB) and activator protein 1 (AP-1) activation, alongside other pathways such as the cyclic GMP-AMP synthase/stimulator of interferon genes pathway, highlighting broader pleiotropic benefits beyond lipid lowering [173,174]. Collectively, these findings establish the critical proof of concept that RNAi could achieve specific, long-lasting LDL-C lowering, thereby validating the use of PCSK9 as a target and directly enabling the development of clinical RNAi therapeutics [175].

Targeting *APOC3* is a paradigm of genetics-driven drug discovery, as human loss-of-function variants are linked to decrease TGs and lower cardiovascular risk [176–178]. Preclinical studies in transgenic mouse models expressing human APOC3 demonstrated that both ASOs and GalNAc-conjugated siRNAs efficiently knocked down hepatic APOC3, leading to a more effective reduction in plasma APOC3 [179]. Subsequent research focused on optimizing the siRNA chemical scaffold to increase durability and tolerability. A systematic structure–activity relationship study identified a novel, long-acting APOC3 siRNA with minimal chemical modifications. This optimized candidate demonstrated long duration of action and an excellent safety profile across species, with comparative analysis suggesting the potential for less frequent dosing than earlier clinical-stage candidates [180]. The translational importance of this target has been further demonstrated in a novel mouse model of combined hyperlipidemia, where therapeutic lipid lowering successfully reduced atherosclerotic plaque burden, providing a robust system for evaluating future APOC3-targeting therapies [181]. Finally, quantitative systems pharmacology modeling of a GalNAc-conjugated APOC3 siRNA has elucidated the key in vivo delivery processes responsible for its sustained pharmacodynamics, successfully enabling PK/PD translation from animals to humans [182].

Increased Lp(a) is a genetically determined risk factor previously inaccessible to pharmacotherapy. A preclinical proof of concept was established with olpasiran, a GalNAc-conjugated siRNA. In transgenic mice and cynomolgus monkeys, a single dosage produced a dose-responsive decrease in plasma Lp(a), achieving a >80% decrease that was sustained for 5 to 8 weeks, demonstrating the potent and durable silencing achievable through this modality [183,184]. This RNAi approach has been further validated with the GalNAc-conjugated siRNA SLN360. In primary hepatocytes, SLN360 potently and selectively silences *LPA* mRNA. In cynomolgus monkeys, a single subcutaneous dose produced a dose-dependent, persistent drop in serum Lp(a) of up to 95%, with effects lasting ≥9 weeks. The study established a minimally effective dose (0.3 mg/kg), highlighting the potent and durable pharmacology achievable with this platform [185]. Further optimization of the siRNA platform has yielded candidates with extended durability. The siRNA RN026 demonstrated profound and sustained Lp(a) lowering in preclinical models. In transgenic mice expressing human *LPA*, a single dose lowered the serum Apo(a) concentration by 98% for more than 6 weeks. In nonhuman primates, a single 2-mg/kg dose achieved a 99% peak reduction in Lp(a), with a 95% reduction maintained at 99 d postdose. RNA sequencing analysis indicated minimal off-target risk, and the candidate was well tolerated in toxicology studies. The preclinical profile suggests the potential for annual dosing intervals in humans, representing a substantial advance in the therapeutic targeting of Lp(a) [186].

In addition to targeting individual lipoproteins, RNAi also enables the silencing of integrative regulators of lipid metabolism. Mechanistic studies in genetic and RNAi-mediated mouse models have demonstrated that ANGPTL3 silencing reduces LDL-C not only by inhibiting lipoprotein lipase but also by a distinct dual mechanism: reducing the hepatic secretion of ApoB-containing lipoproteins and increasing their clearance via the LDLR pathway [187]. This provides a strong scientific basis for its ability to lower multiple atherogenic lipids. Therapeutically, this rationale has been validated with potent RNAi candidates. The GalNAc-conjugated siRNA ANGsiR10 demonstrated remarkable efficacy in dyslipidemic animal models. In transgenic mice with hypertriglyceridemia, a single dose reduced plasma TG by 96%. In a rhesus monkey model of spontaneous dyslipidemia, a single dose achieved a 68% reduction in TGs, with the lipid-lowering effect persisting for up to 15 weeks. Treatment also substantially reduced non-HDL cholesterol levels [188]. Together, this body of work provides definitive evidence that sustained ANGPTL3 silencing with an RNA-targeted therapeutic can achieve profound and durable correction of atherogenic dyslipidemia through multiple mechanisms [187,188].

Targeting hepatic cholesterol synthesis: ACLY siRNA, the RNAi platform is also being leveraged to target fundamental intracellular metabolic enzymes, expanding its reach beyond secreted regulators. A prime example is ATP-citrate lyase (ACLY), a key enzyme upstream of HMG-CoA reductase in the cholesterol biosynthesis pathway. The clinical validation of ACLY as a target was established by the small-molecule inhibitor bempedoic acid [189,190]. Translating this pharmacology into the RNAi domain, a GalNAc-conjugated siRNA targeting hepatic ACLY, aclysiran, has demonstrated compelling preclinical proof of concept. In a murine model of atherosclerosis, monthly administration of aclysiran achieved potent and sustained (~1 month) knockdown of hepatic ACLY, leading to substantial reductions in plasma LDL-C and TGs and attenuation of atherosclerotic plaque burden comparable to that of daily oral bempedoic acid. Importantly, aclysiran operates independently of the ACSVL1 activation required by the small-molecule prodrug, potentially offering a more consistent therapeutic response and bypassing mechanisms associated with muscle toxicity. This approach mechanistically recapitulates the benefits of pharmacological ACLY inhibition but confers an extended dosing interval intrinsic to the siRNA platform [191].

By enabling durable silencing of a critical intracellular enzyme, aclysiran exemplifies the expansion of RNA-targeted therapeutics into direct modulation of cytoplasmic metabolic pathways, offering a novel, long-acting strategy to suppress de novo cholesterol synthesis for patients with statin intolerance or residual cardiovascular risk. Collectively, these preclinical proofs of concept across multiple targets validate the RNAi platform, and their future translational progress will be accelerated by employing advanced experimental models such as vessels-on-a-chip, tissue-engineered vessels, and models of vulnerable plaques that better recapitulate human atherosclerotic pathophysiology [192].

## RNA-Targeted Therapeutics for Lipid-Metabolic Disorders: Bedside Evidence

**Fig. 4.**
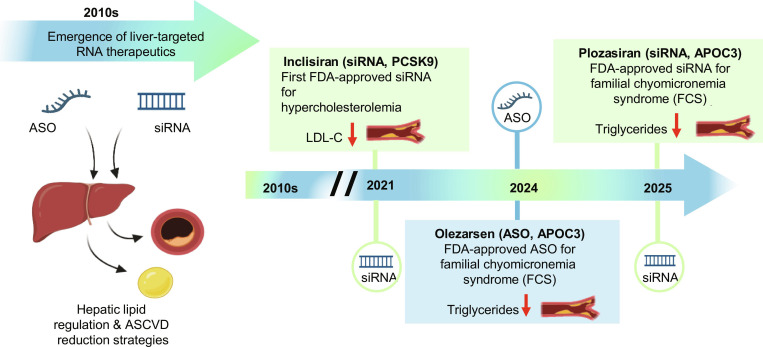
Timeline of key FDA approvals for liver-targeted RNA therapeutics in lipid regulation (2010s to 2025). This schematic timeline illustrates the emergence and clinical translation of 2 major classes of RNA therapeutics, ASOs and siRNAs, which target hepatic genes for the management of dyslipidemia and associated cardiovascular risk.

The strong mechanistic and translational foundation established by preclinical studies has enabled rapid clinical advancements in RNA-targeted therapeutics for lipid-driven atherosclerosis (Fig. 4). By targeting genetically validated drivers of dyslipidemia, these agents have demonstrated profound, durable lipid lowering with infrequent dosing schedules, addressing major limitations of conventional therapies.

### PCSK9 siRNA: Inclisiran

The successful development of PCSK9 inhibitors, from seminal genetic discoveries to large cardiovascular outcomes trials, has established this class as a potent LDL-C-lowering strategy with an excellent safety profile, paving the way for RNA-targeted approaches such as inclisiran. The evolution of PCSK9-targeted therapies now includes RNA interference, oral inhibitors, and gene therapy as emerging modalities [193]. Inclisiran (Leqvio) is an FDA-approved siRNA therapy for ASCVD and heterozygous FH [194]. It works by silencing PCSK9, an enzyme that breaks down LDLRs, thereby lowering LDL cholesterol [195]. Phase 1 trials revealed a 40% to 50% decrease in LDL-C, which was confirmed in the larger ORION-1 phase 2 study (NCT02597127). In that trial, 2 300-mg subcutaneous doses given 90 d apart produced the best results, with 48% of patients achieving very low LDL-C levels by day 180 [196]. These findings informed phase 3 trials (ORION-10 [NCT03399370] and ORION-11 [NCT03400800]) in cardiovascular patients, including those with high LDL-C despite maximum statin therapy, where LDL-C was reduced by approximately 50% through day 510 [141]. The subsequent VICTORION-2P cardiovascular outcomes trial (NCT05030428) in patients with established ASCVD is ongoing and expected to determine its impact on MACE [197].

The efficacy and safety of inclisiran have now been extended to pediatric populations. In the phase 3 ORION-16 trial involving adolescents with heterozygous FH, inclisiran 300 mg administered subcutaneously on days 1, 90, and 270 reduced LDL cholesterol by 28.5% compared with placebo at day 330, with sustained effects over 2 years and no treatment-related serious adverse events (NCT04652726) [198]. This efficacy is supported by real-world evidence. A meta-analysis of real-world studies demonstrated an average LDL-C reduction of 42.8%, with greater reductions observed when inclisiran was combined with statin therapy (45.7%) than when it was used as monotherapy [199]. Other siRNA therapies in development include ARO-APOC3, which targets APOC3 to lower TGs and has received FDA fast-track status for severe hypertriglyceridemia and related conditions. Similarly, ARO-ANG3, which is now in phase 2 trials, silences ANGPTL3 to treat mixed dyslipidemia [200]. Additional candidates, such as SLN360 (phase 2) and olpasiran (phase 1), are being studied to reduce Lp(a) [201]. Thus, RNA interference has matured from genetic insight into a robust therapeutic platform, exemplified by the approved siRNA inclisiran for lowering LDL-C, with a promising pipeline extending its durable silencing strategy to other key lipid regulators, such as APOC3, ANGPTL3, and Lp(a), to address the multifaceted nature of residual CVD risk.

### ApoC3-targeted therapies

ApoC3 is a genetically validated, high-value target for reducing residual cardiovascular risk [32]. ApoC3 operates through a parallel pathway to ANGPTL3 and inhibits lipoprotein lipase, impairing the removal of TG-rich lipoproteins (chylomicrons, VLDL, and remnants). This is confirmed by human genetics, where loss-of-function mutations lead to low TG levels and reduced cardiovascular risk [202]. Volanesorsen, an ASO that targets ApoC3, was approved by the FDA in 2019 [203] and markedly reduced plasma TGs and VLDL in individuals with severe hypertriglyceridemia (NCT02300233) and FCS (NCT02211209) [204,205]. However, its clinical use has been restricted by adverse effects, including thrombocytopenia, prompting the development of next-generation agents [203,204]. Olezarsen, a next-generation, GalNAc-conjugated ASO whose promising pharmacology was established in a phase 1/2a trial (NCT02900027), which reported reductions of up to 92% in APOC3 and 77% in TGs [206], was FDA approved in December 2024 for adults with FCS [207]. It inhibits hepatic ApoC3, enhancing TG clearance. The inhibition of hepatic ApoC3 synthesis enhances TG clearance. In the phase 3 BALANCE trial, olezarsen reduced TG levels by approximately 60% in patients with FCS and markedly lowered the incidence of acute pancreatitis events (NCT04568434) [208].

In addition to ASO development, siRNA approaches targeting APOC3 have also demonstrated substantial promise. Plozasiran (ARO-APOC3), a hepatocyte-targeted GalNAc-conjugated siRNA, substantially reduced TG levels in a 48-week phase 2b trial of individuals with mixed hyperlipidemia. At week 24, plozasiran achieved dose-dependent reductions in fasting TGs of 50% to 62% with quarterly dosing and 44% with half-yearly dosing compared with placebo. This trial confirmed that durable APOC3 silencing via RNAi is a potent therapeutic strategy with flexible dosing intervals (NCT04998201) [209]. The efficacy of this siRNA strategy in a more severe population was demonstrated in the SHASTA-2 trial (NCT04720534) in individuals with severe hypertriglyceridemia (TGs 500 to 4,000 mg/dl). Here, plozasiran induced potent, dose-dependent reductions in APOC3 (up to −77%) and TGs (up to −57% placebo-adjusted) at 24 weeks, with 91% of treated individuals achieving TG levels below the 500-mg/dl acute pancreatitis risk threshold. Notably, while marked increases in LDL-C were observed at the highest dose, this increase was accompanied by no change in ApoB and substantial reductions in atherogenic non-HDL-C and remnant cholesterol, suggesting a shift toward larger, more cholesterol-enriched LDL particles. The safety profile was favorable across all doses (NCT04720534) [210]. On the basis of this evidence, the FDA approved plozasiran (Redemplo) on 2025 November 18, for adults with FCS to reduce very high TGs, marking the first siRNA treatment approved for this disorder on the basis of considerable decrease in TGs and pancreatitis risk demonstrated in the phase 3 PALISADE trial (NCT05089084) [211]. A recent meta-analysis of 8 randomized controlled trials confirmed that APOC3-targeted siRNA therapies substantially reduce TGs by 52%, with a concomitant increase in HDL-C of 40.9%, supporting their efficacy in managing hypertriglyceridemia [212].

Collectively, the dual approvals of olezarsen and plozasiran demonstrate the successful translation of human genetics into 2 distinct, long-acting RNA-targeted therapeutic platforms, offering powerful new options for normalizing TGs, mitigating pancreatitis risk, and reducing residual cardiovascular risk.

### Lp(a)-targeted therapies

Lp(a) is a genetically determined and largely statin-resistant risk factor for ASCVD [213–215], making it an ideal candidate for RNA-targeted intervention. Existing lipid-lowering therapies, including PCSK9 inhibitors, provide only modest Lp(a) reduction, falling short of the substantial lowering required for meaningful risk mitigation. Reflecting the growing recognition of Lp(a) as a critical cardiovascular risk factor, the 2026 ACC/AHA Multisociety Guideline on the Management of Dyslipidemia now recommends universal Lp(a) testing at least once in all adults (class 1 recommendation). Levels ≥125 nmol/l (≥50 mg/dl) are associated with a 1.4-fold increased ASCVD risk, while levels ≥250 nmol/l confer a >2-fold increased risk [216], underscoring the urgent need for effective Lp(a)-lowering therapies. This unmet need has accelerated the development of RNA-targeted agents. Pelacarsen, a GalNAc-conjugated ASO made to inhibit hepatic Apo(a) synthesis, demonstrated the feasibility of this approach. In a phase 2b study, pelacarsen achieved robust Lp(a) reductions of ≥80%, with a favorable safety profile, and it is currently being evaluated for cardiovascular outcomes in the phase 3 Lp(a) HORIZON trial (NCT04023552). By providing critical proof of concept, pelacarsen has validated Lp(a) as a druggable target and paved the way for next-generation therapies [217,218]. Consistent with this, subsequent trial data reported even greater dose-dependent reductions of up to 97% [219].

While pelacarsen was validated as the target, the subsequent focus shifted to the siRNA platform, which enables even more profound and durable Lp(a) suppression through the catalytic RNA interference mechanism. Olpasiran, a GalNAc-conjugated siRNA, demonstrated transformative Lp(a)-lowering effects in the phase 2 OCEAN(a)-DOSE trial, with doses ≥75 mg every 12 weeks reducing Lp(a) by >95% (NCT04270760) [220]. In addition to this quantitative reduction, olpasiran also potently reduced the number of oxidized phospholipids on apolipoprotein B (OxPL-ApoB) by up to 93% in a dose-dependent manner [221]. These findings suggest that therapy not only lowers Lp(a) mass but also may directly mitigate its pathogenicity. Notably, these reductions in OxPL occurred without marked changes in systemic inflammatory markers such as high-sensitivity C-reactive protein or interleukin-6 in the trial timeframe. The consistency of this pharmacologic profile has been demonstrated globally, with a phase 1 study in a Chinese population confirming that single doses produced profound reductions (up to −99.2%) and a favorable safety profile aligning with other ethnic groups [222].

Lepodisiran is an investigational, long-acting GalNAc-conjugated siRNA designed to inhibit hepatic LPA transcription. Clinical data demonstrate its potent efficacy, with dose-dependent Lp(a) reductions of up to 97% and a durable effect that positions it as a promising candidate for annual or biannual dosing [223]. In a phase 1 single ascending-dose trial (NCT04914546), single subcutaneous doses produced profound, dose-dependent reductions in Lp(a), with the highest dose (608 mg) achieving a median maximal reduction of −97% and sustaining a −94% reduction at 337 d, alongside a favorable safety profile [224]. These promising results were strongly confirmed and refined by the larger, placebo-controlled phase 2 ALPACA trial (NCT05565742). Here, a single 400-mg dose produced a time-averaged Lp(a) reduction of −93.9% from day 60 to 180, and a 2-year regimen maintained a −94.8% reduction through 1 year, confirming its profile as a promising candidate for annual or biannual dosing [225].

Another potent GalNAc-conjugated siRNA, zerlasiran (SLN360), produces robust, sustained Lp(a) lowering. Initial human data from a phase 1/2 trial (NCT04606602) demonstrated its foundational promise, where 2 doses achieved near-complete median Lp(a) reductions of up to −99%, with effects persisting markedly for over 200 d postdose [226]. These results were confirmed and refined in a dedicated phase 2 trial (NCT05537571) in patients with ASCVD. Here, multiple dosing regimens (300 to 450 mg every 16 to 24 weeks) produced robust, time-averaged Lp(a) reductions of >80% over 36 weeks, confirming its profile for quarterly to biannual administration [227]. RNA-targeted therapies have shifted Lp(a) from an untreatable risk factor to a pharmacologically modifiable target, with potent siRNAs now undergoing definitive trials to confirm their clinical benefit. The rapid progress in this field has been recently reviewed, confirming that multiple RNA-targeted agents consistently achieve ≥80% Lp(a) reductions in phase 2 trials, with definitive cardiovascular outcome studies now underway [228].

### ANGPTL3-targeted therapies

The inhibition of ANGPTL3 via RNA therapeutics represents a novel strategy for managing complex dyslipidemia, with ASOs and siRNAs having distinct clinical narratives. Vupanorsen (ANGPT3-LRx), a GalNAc-conjugated ASO designed to inhibit hepatic ANGPTL3 synthesis, was evaluated in the phase IIb TRANSLATE-TIMI 70 trial. In statin-treated patients with mixed dyslipidemia, vupanorsen reduced non-HDL cholesterol by approximately 25% and TGs by 41% to 57%. However, it produced only modest reductions in LDL cholesterol and ApoB. The trial also raised safety concerns, including elevated liver enzymes and dose-dependent increases in hepatic fat content up to 76%, which led to the discontinuation of its development (NCT04516291) [229]. A dedicated analysis of the TRANSLATE-TIMI 70 trial further elucidated Vupanorsen’s mechanism, demonstrating that ANGPTL3 inhibition potently reduced atherogenic remains of cholesterol by 42% to 59% and VLDL-C by 52% to 67%, with the magnitude of reduction directly correlated with the degree of ANGPTL3 silencing [230].

Zodasiran, a GalNAc-conjugated siRNA, targets hepatic ANGPTL3 expression. Its efficacy and safety were assessed in a 24-week, phase IIb ARCHES-2 trial involving 204 individuals with mixed hyperlipidemia (NCT04832971) [231]. Zodasiran treatment produced dose-dependent reductions, with the 200-mg dose lowering TGs by >50%, non-HDL cholesterol by 36%, ApoB by 22%, and LDL cholesterol by 20%. Unlike the ANGPTL3-targeted ASO vupanorsen, zodasiran did not elevate liver enzymes or fat. However, it was associated with transient increases in glycated hemoglobin in some individuals with pre-existing diabetes at the highest dose. The development of zodasiran for mixed hyperlipidemia has been promoted by Arrowhead in favor of its APOC3-targeted candidate, plozasiran [209]. Beyond mixed hyperlipidemia, zodasiran has shown promise in homozygous familial hypercholesterolemia (HoFH), a population with limited treatment options. The phase 2 GATEWAY trial reported LDL cholesterol reductions of approximately 35.7% to 39.9% at month 6 in patients with HoFH, with even greater reductions (55.8%) observed in those also receiving concomitant PCSK9 inhibitor therapy, supporting its potential in this high-risk population [232].

Solbinsiran, a GalNAc-conjugated siRNA, an ANGPTL3 inhibitor, progressed to a phase II trial of interest following its promising evaluation in earlier preclinical and phase I studies (NCT04644809) [233]. The durability and safety of solbinsiran were evaluated in PROLONG-ANG3 (NCT05256654), a double-blind, randomized, placebo-controlled phase II trial. The study revealed a dose-dependent effect on the primary endpoint. Compared with the placebo, the 400-mg dose administered on days 0 and 90 produced a significant reduction in ApoB on day 180 (−14.3%, *P* = 0.0085), whereas the effects of the 100- and 800-mg doses did not reach statistical significance. Treatment was generally well tolerated, with a lower incidence of adverse events in the solbinsiran group (44% to 60%) than in the placebo group (65%). The study concluded that solbinsiran 400 mg effectively reduces ApoB and is well tolerated, although its effect on cardiovascular outcomes remains to be determined [234]. A recent editorial commentary on the PROLONG-ANG3 trial highlights the dual effect of solbinsiran on both TG-rich remnants and LDL particles, its favorable hepatic safety profile with potential benefits on hepatic steatosis, and the practical advantage of quarterly subcutaneous dosing, while noting that the inconsistent dose response and short follow-up limit long-term conclusions [235]. Thus, the therapeutic targeting of ANGPTL3 has evolved from a hepatotoxic ASO to safer, durable siRNA candidates, which now await definitive outcome trials to confirm their clinical potential in cardiovascular risk reduction. Meta-analytic data further demonstrated that ANGPTL3-targeted siRNAs provide broader lipid modulation, reducing TGs by 52% and LDL-C by 13.2%, while also decreasing HDL-C by approximately 20%, a differential effect distinct from APOC3-targeted agents [212]. Collectively, the clinical development and regulatory status of leading siRNA and ASO therapies for dyslipidemia are summarized in Table 1.

**Table 1.** Clinical development status of siRNA and ASO therapies for dyslipidemia

Drug name	Target	Type	Current status	Clinical trial (NCT)	Company
Inclisiran	PCSK9	siRNA	FDA approved (2021) for hypercholesterolemia	Multiple ORION and VICTORION trials	Novartis Pharmaceuticals
Volanesorsen	APOC3	ASO	Rejected by FDA; conditionally approved in EU for FCS (Phase III)	(NCT02211209) [204]	Ionis Pharmaceuticals and Akcea Therapeutics
Olezarsen	APOC3	ASO	FDA approved for FCS (2024); Investigational for severe hypertriglyceridemia (Phase II)	CORE (NCT05079919) and CORE-2 (NCT05552326) [241]; CORE-OLE (NCT05681351)	Ionis Pharmaceuticals
Plozasiran	APOC3	siRNA	FDA approved for FCS (2025); SHTG and MUIR-3 trials ongoing	(NCT05089084) [211]; (NCT06347003); (NCT06347133)	Arrowhead Pharmaceuticals
Pelacarsen	Lp(a)	ASO	Phase III (Investigational)	(NCT04023552) [218]	Novartis Pharmaceuticals
Olpasiran	Lp(a)	siRNA	Phase III (Investigational)	(NCT05581303)	Amgen
Lepodisiran	Lp(a)	siRNA	Phase III (Investigational)	(NCT06292013)	Eli Lilly and Company
Zerlasiran	Lp(a)	siRNA	Phase I (Investigational)	(NCT04606602) [226]	Silence Therapeutics
Vupanorsen	ANGPTL3	ASO	Discontinued (2022)	-	Pfizer/Ionis
Zodasiran	ANGPTL3	siRNA	Phase III	(NCT07037771)	Arrowhead Pharmaceuticals
Solbinsiran	ANGPTL3	siRNA	Phase II	(NCT07269210)	Eli Lilly & Company

siRNA, small interfering RNA; ASO, antisense oligonucleotide; FDA, Food and Drug Administration; EU, European Union; FCS, familial chylomicronemia syndrome; SHTG, severe hypertriglyceridemia; PCSK9, proprotein convertase subtilisin/kexin type 9; APOC3, apolipoprotein C-III; Lp(a), lipoprotein(a); ANGPTL3, angiopoietin-like protein 3

## Concluding Remarks and Future Perspective

**Fig. 5.**
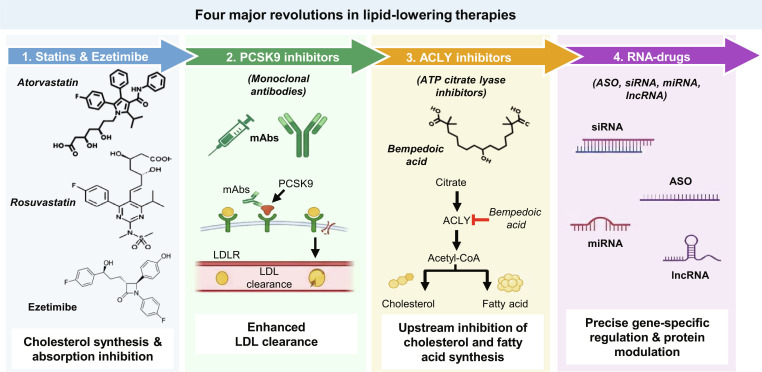
Four major revolutions in lipid-lowering therapies. The figure depicts the chronological and mechanistic progression of lipid-lowering strategies. The first revolution (statins and ezetimibe) reduces cholesterol synthesis and its absorption. The second revolution (PCSK9 inhibitors) uses monoclonal antibodies to enhance LDL receptor clearance. The third revolution (ACLY inhibitors, e.g., bempedoic acid) blocks ATP-citrate lyase upstream of statins, lowering cholesterol and fatty acids with liver-specific action. The fourth revolution (RNA-drugs) encompasses ASOs, siRNAs, anti-miRs, and lncRNA modulators, enabling durable gene silencing and precise regulation of atherogenic targets. Together, these revolutions illustrate a shift from broad metabolic inhibition to gene-specific, long-acting therapies for dyslipidemia.

The evolution of lipid-lowering therapy can be viewed as a series of revolutionary advances (Fig. 5). Statins and ezetimibe, which inhibit cholesterol synthesis and absorption, respectively, represented the first revolution, dramatically reducing LDL-C and cardiovascular events. The second revolution came with PCSK9 monoclonal antibodies, which provided profound LDL-C lowering on top of statins. The third revolution was marked by ACLY inhibitors such as bempedoic acid, offering an oral alternative for statin-intolerant patients. Now, RNA-targeted therapeutics, including siRNAs (e.g., inclisiran and plozasiran), ASOs (e.g., olezarsen and pelacarsen), anti-miR agents, and mRNA-LNP-based in vivo base editing (e.g., VERVE-101/102), constitute the fourth revolution. These modalities achieve durable gene silencing or permanent genetic correction with infrequent dosing, promising to address residual cardiovascular risk more effectively than ever before.

Moving beyond the transient modulation of proteins by small molecules, RNA platforms, specifically ASOs and siRNAs, enable precise, durable silencing of disease-causing genes at their transcriptional origin. As this review has detailed, the clinical validation of this approach, exemplified by inclisiran for PCSK9, is now being powerfully extended to other genetically validated targets, such as APOC3, Lp(a), and ANGPTL3, with agents such as plozasiran, olezarsen, olpasiran, and lepodisiran demonstrating profound, long-lasting reductions in key atherogenic lipids. The success of this first generation of cardiovascular RNA-targeted drugs rests on the foundational innovation of GalNAc conjugation, which facilitates efficient, hepatocyte-specific delivery via the ASGPR. This technology has transformed the therapeutic landscape for hepatic targets, offering the dual benefit of exceptional efficacy and infrequent dosing schedules (quarterly to biannually), which promises to overcome the adherence challenges that limit the real-world effectiveness of daily oral therapies. However, the very success of this liver-centric strategy highlights a critical frontier for the field.

**Fig. 6.**
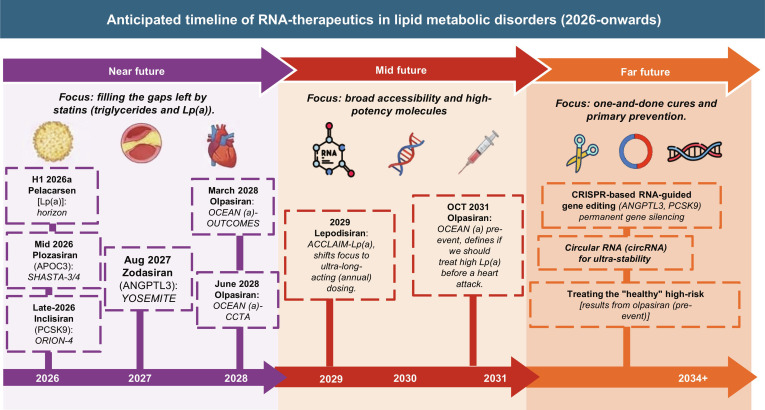
Anticipated timeline of RNA-targeted therapeutics in lipid metabolic disorders. Schematic representation of the projected evolution of RNA-based therapeutics from 2026 to beyond 2034, highlighting the transition from current lipid-lowering strategies to precision prevention and potential curative approaches. In the near term (2026 to 2028), RNA therapies expand from rare disorders to mainstream management of dyslipidemia, with clinical validation of targets such as PCSK9, apolipoprotein C-III (APOC3), angiopoietin-like protein 3 (ANGPTL3), and lipoprotein(a) [Lp(a)] through key trials including HORIZON, SHASTA, ORION-4, and OCEAN(a). The mid-term phase (2029 to 2031) emphasizes improved accessibility, higher potency, and ultra-long-acting therapies, alongside a shift toward early intervention and primary prevention in genetically at-risk populations. In the far future (2031 to 2034+), advances in gene-editing technologies, including CRISPR-based approaches and circular RNA platforms, may enable permanent silencing of lipid-associated genes and transition the field from chronic treatment to one-time curative strategies. Collectively, this timeline illustrates the progressive shift from LDL-C-centric therapy to multitarget lipid regulation, precision medicine, and, ultimately, disease prevention and cure.

The next phase of RNA-targeted therapeutics for lipid-driven ASCVD may extend beyond hepatic lipid regulation toward the modulation of vascular inflammation and plaque biology. Looking further ahead, while GalNAc-conjugated RNAs have established efficient and durable liver targeting for genes such as PCSK9, APOC3, ANGPTL3, and Lp(a), addressing residual cardiovascular risk will require extrahepatic delivery to immune and vascular cells that drive plaque progression. Emerging nanoparticle platforms, particularly LNPs, are being explored for their potential to deliver nucleic acid therapeutics directly to hepatocytes and vascular cells involved in lipid metabolism [126,236]. LNPs offer a versatile platform capable of delivering not only siRNA to silence disease-causing genes [e.g., targeting PCSK9, ANGPTL3, APOC3, or Lp(a)] but also mRNAs to express therapeutic proteins, opening new avenues for treating dyslipidemia and atherosclerosis [237,238]. The 2026 ACC Scientific Statement on Gene Editing Therapy in Cardiovascular Disease underscores the clinical readiness of these approaches, particularly for hepatically expressed targets such as PCSK9, ANGPTL3, and Lp(a), while also highlighting the need for long-term safety monitoring, equitable access, and ethical implementation [131]. These delivery advances will enable rational combination therapies, in which RNA-targeted agents are integrated with statins or other lipid-modifying interventions to achieve synergistic and durable risk reduction. However, efficient and safe extrahepatic delivery remains a major technical challenge, and most strategies are currently in preclinical or early translational stages. Complementing these delivery efforts, recent systematic screens have prioritized novel, causal targets for CVD, thereby mapping a concrete pipeline for the next generation of RNA-targeted therapies [239]. A recently published comprehensive review further highlights the rapidly expanding landscape of mRNA-based biotechnologies for CVDs, including mRNA modifications, delivery platforms, genome and epigenomic editing, and chimeric antigen receptor engineering for immune cell therapy, with preclinical and clinical applications in hypercholesterolemia, atherosclerosis, cardiac injury, fibrosis, and amyloidosis [240]. In parallel, targeting inflammatory transcriptional programs within plaque-resident cells could potentially expand RNA-targeted therapeutics beyond lipid lowering toward modulation of vascular disease processes, potentially positioning them as an important component of future precision therapies of ASCVD. A projected timeline of anticipated milestones for RNA-targeted therapeutics in lipid metabolic disorders is shown in Fig. 6. We can envisage more RNA-targeted drugs against hypercholesterolemia and ASCVD will emerge in the decades to come.
